# Factors Associated With Mental Health Outcomes Across the Lifespan

**DOI:** 10.5888/pcd22.250371

**Published:** 2025-12-18

**Authors:** Leonard Jack

**Affiliations:** 1 Preventing Chronic Disease, Office of the Director, National Center for Chronic Disease Prevention and Health Promotion, Centers for Disease Control and Prevention, Atlanta, Georgia

Research describing the relationship between mental and physical health has expanded during the past decade (1). According to the Substance Abuse and Mental Health Services Administration, components of mental health include an individual’s emotional, psychological, and social well-being (1). Mental health influences day-to-day feelings, activities, and functions that range from a sense of social connection, participation in physical activity, eating habits, work productivity, academic performance, and self-esteem (2,3). Mental health issues can affect anyone at any age (3). For these reasons, gaining insight into factors influencing mental health outcomes across the lifespan is imperative.

*Preventing Chronic Disease* (PCD) is committed to publishing timely research that brings attention to mental, psychological, and emotional health. In 2020, PCD published *Mental Health Is a Global Public Health Issue* (4), a collection of 10 peer-reviewed articles that offered insight into the many pathways through which factors affect psychological well-being (4). Today, we are pleased to release a new collection, *Factors Associated With Mental Health Outcomes Across the Lifespan*, with the goal of increasing access to research that can help shape tailored public health and clinical responses to improve mental health outcomes. Five peer-reviewed articles in this collection address the following topics: factors associated with children not receiving mental health services; trends in mental, behavioral, and developmental disorders among children and adolescents; the association between diet and mental health outcomes among adolescents; nurse-led mental health interventions for college students; and mental health symptoms and receipt of mental health care among adults diagnosed with kidney disease.

Meng and Wiznitzer emphasize that many mental health disorders begin in early childhood (5). These authors report findings from a national sample of 46,424 children aged 2 to 8 years from the National Survey of Children’s Health (2021 and 2022) to estimate the prevalence of having a mental health disorder and identify factors associated with young children not receiving mental health care when needed (6). According to the authors, 19.0% of US children aged 2 to 8 years had 1 or more mental health disorders, and 45.8% of those with a mental health disorder did not receive mental health services when needed. The article offers insights into possible ways to address this problem, including establishing patient-centered communications and enhancing patients’ experiences with health care providers.

Leeb and colleagues used data from the National Survey of Children’s Health (2016–2021) to examine trends in parent-reported lifetime childhood mental, behavioral, and developmental disorders among children aged 3 to 17 years (6). The authors reported that the prevalence of these disorders among children increased from 25.3% in 2016 to 27.7% in 2021. These researchers recommended improving pediatric mental health training for health care providers, developing clinical care interventions, and increasing access to mental health services.

The association between diet and mental health among children has received increasing attention in the past 5 years (7,8). In this PCD collection, an article by Dabravolskaj and colleagues describe the association between diet and mental health outcomes among 13,887 adolescents aged 14 to 18 years in Canada (9). Their findings indicated that consumption of sugar-sweetened beverages was associated with greater severity of depressive and anxiety symptoms and that consumption of fruits and vegetables was positively associated with psychological well-being. The authors concluded that diet should be an important component of a comprehensive approach for achieving psychological health.

College students experience high levels of stress and worry due to factors that typically accompany their transition into adulthood: academic pressures, identity issues, financial difficulties, struggles with interpersonal relationships, isolation, and increased responsibilities (10). An article in PCD’s collection describes a systematic review of nurse-led interventions conducted by Russell and colleagues to identify the various approaches used by nurses to enhance mental well-being among college students. The review described 16 articles that met inclusion criteria from institutions in North America, Europe, and Asia (11). The authors reported that nurse-led interventions, including experimental, quality improvement, and educational approaches, offer promising ideas for resolving mental health problems among college students.

Living with a chronic condition affects both physical health and mental health (12–16). In this PCD collection, Villarroel and Wang used data from the 2021 National Health Interview Survey to estimate mental health symptoms and services among adults living with kidney disease (16). These authors narrowed their research focus to adults with kidney disease and more specifically, advanced kidney disease. They analyzed data on serious psychological distress, current symptoms of anxiety and depression, use of prescription medication for these disorders, and receipt of counseling. Findings showed that individuals with kidney disease had a higher prevalence of poor mental health and receipt of mental health care than those without kidney disease.

Mental health status influences physical, emotional, and psychological outcomes across the lifespan. Authors of articles included in this PCD collection suggest the need for more research to identify pathways to improve mental health outcomes for individuals living with less-than-optimal mental health along with identifying ways that families, health care providers, and communities can support them. More training is needed for health professionals working on the frontlines to treat individuals living with mental health disorders and health professionals working to establish and enhance delivery of interventions within and outside clinical settings (17,18). PCD will continue to encourage those working in this important, but less published, area of study to submit their work to the journal. Publishing articles that describe such work will help the journal achieve its goal of increasing awareness that mental health is a global public health issue that can be addressed at various touchpoints throughout the lifespan.
